# Post-infection brain atrophy accelerates cognitive and molecular changes underlying dementia

**DOI:** 10.1186/s13195-025-01924-2

**Published:** 2025-12-05

**Authors:** Michael R. Duggan, Pyry N. Sipilä, Zhijian Yang, Junhao Wen, Guray Erus, Murat Bilgel, Alexandria Lewis, Abhay Moghekar, Christos Davatzikos, Susan M. Resnick, Mika Kivimäki, Keenan A. Walker

**Affiliations:** 1 Laboratory of Behavioral Neuroscience, National Institute On Aging, National Institutes of Health, Baltimore, MD USA; 2 Department of Public Health, Clinicum, University of Helsinki, Helsinki, FI USA; 3 Artificial Intelligence in Biomedical Imaging Laboratory, Perelman School of Medicine, University of Pennsylvania, Philadelphia, USA; 4 Laboratory of AI and Biomedical Science, Columbia University, New York, NY USA; 5 Department of Neurology, Johns Hopkins University School of Medicine, Baltimore, MD USA; 6 Brain Sciences, University College London, London, UK

**Keywords:** Infection, Longitudinal, Neuroimaging, Biomarkers, Cognitive decline

## Abstract

**Background:**

Infections have been associated with a greater risk of Alzheimer’s disease and related dementias (ADRD), but it is unclear how infections influence structural brain patterns over time, and whether post-infection brain atrophy can accelerate cognitive decline and molecular changes underlying dementia.

**Methods:**

Using the Baltimore Longitudinal Study of Aging (BLSA; *n* = 793; mean age = 70.1), we examined how infections relate to longitudinal changes in machine learning-derived, 3 T-MRI neuroimaging signatures, and leveraged the UK Biobank (UKB; 1,120; mean age = 62.9 yrs) to externally validate infection-brain atrophy relationships. Using the BLSA, we also asked if infection history and infection-related brain volume loss were associated with cognitive decline, amyloid-beta PET, and ADRD plasma biomarker trajectories (Aβ_42/40_, pTau-181, NfL, GFAP).

**Results:**

We detected accelerated parieto-temporal atrophy in BLSA participants with a history of upper respiratory tract, bacterial, and urinary tract infections (*p* < 0.05), as well as influenza and skin/subcutaneous infections (FDR *p* < 0.05). After demonstrating their associations with longitudinal neuroimaging signatures in the UKB and prevalent dementia in the BLSA, we found that infections were related to a greater burden of ADRD plasma biomarkers and accelerated rates of cognitive decline in BLSA participants. Integrating longitudinal brain scans, cognitive assessments, and plasma biomarker measurements, we identified infection-related changes in verbal memory and NfL that were more prominent among BLSA participants who experienced greater post-infection brain atrophy.

**Conclusion:**

Along with demonstrating that infections mediate clinically relevant brain atrophy patterns, these findings highlight the consequences of post-infection brain volume loss on longitudinal neurocognitive outcomes and extend our understanding of the biological basis by which infections may contribute to neurodegeneration.

**Supplementary Information:**

The online version contains supplementary material available at 10.1186/s13195-025-01924-2.

## Introduction

A history of infections has been associated with increased risk of Alzheimer’s disease and related dementias (ADRD) [1], but additional investigations are warranted to elucidate how infections impact longitudinal changes in brain structure, and whether infection-related brain atrophy can influence cognitive decline and molecular changes underlying dementia. Given that both neurotrophic and systemic microbial exposures can influence brain health by reshaping host biological processes (e.g., through chronic inflammation, disruption of blood brain barrier integrity, etc.,), deconvoluting the mechanisms by which infections influence the development of cognitive impairment is critical for understanding their pathophysiological roles in the AD cascade (i.e., increased amyloid and tau burden) as well as in broader neurodegenerative pathways [2]. For example, decreases in gray matter thickness and total brain volume have been reported following SARS-CoV-2 infection [3], and we recently showed reductions in regional brain volumes associated with symptomatic infections [4, 5]. In contrast, some studies have found no associations between herpetic infections and changes in whole brain atrophy, and other reports suggest aviremic people living with HIV may not show brain volume loss [6–8]. Furthermore, it remains unclear if infection-related brain atrophy is evident in structurally distinct and clinically relevant patterns of neurodegeneration.

Machine learning-derived neuroimaging signatures provide a unique opportunity to address this gap in knowledge, because they capture the co-expression of multi-dimensional brain atrophy patterns that predict age-related clinical traits and neurodegenerative disease risk in large cohort studies, including clinical progression and dementia diagnosis [9]. Although we and others have documented cross-sectional associations of infections with cognitive functioning and ADRD plasma biomarkers [4, 10, 11], it also remains unclear how infection history and post-infection structural brain changes might interact to influence the emergence of these ADRD endophenotypes over time.

To address these research questions, we leveraged data from the Baltimore Longitudinal Study of Aging (BLSA) and investigated longitudinal changes in machine learning-derived neuroimaging signatures among participants with different infection diagnoses. For infections associated with accelerated brain atrophy, we further examined whether they were related to increased odds of cognitive impairment in the BLSA and longitudinal changes in machine learning-derived neuroimaging signatures in the UK Biobank (UKB). Among BLSA participants, we also investigated if infection history was associated with cognitive decline and trajectories of ADRD plasma biomarkers, and whether these effects were more pronounced in individuals who experienced greater post-infection brain atrophy. The current study’s overall aim was to identify unique patterns of accelerated brain atrophy among older adults with a history of specific infections and to assess their implications for long-term neurocognitive outcomes.

## Methods

### Study sample

**Fig. 1:**
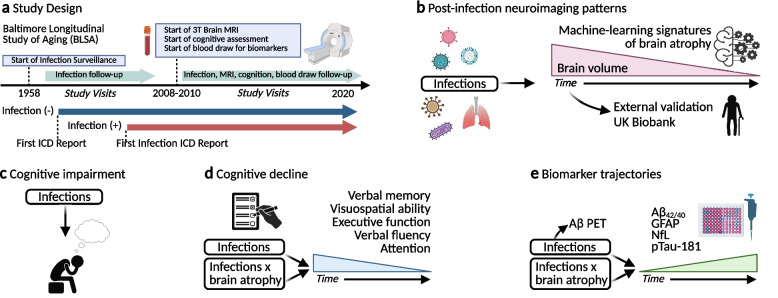
Study design. **a** Baltimore Longitudinal Study of Aging (BLSA) participants were classified according to the presence or absence of an infection diagnosis using ICD codes collected at study visits as early as 1958. Repeated 3 T MRIs began in 2008–2010, at which point serial cognitive assessments and blood draws (i.e., for plasma biomarker measurements) were initiated. Study visits occurred every 1–4 years depending on age (age < 60 years, every 4 years; age 60–79 years, every other year; age ≥ 80 years, annual). **b** Discovery analyses examined whether infection history was associated with longitudinal changes in machine learning-derived neuroimaging signatures of distinct brain atrophy patterns. External validation of infection-related brain volume loss leveraged 3 T-MRI data from the UK Biobank. **c** Further analyses of infections linked to brain atrophy examined their associations with prevalent mild cognitive impairment, dementia, and any form of cognitive impairment. **d** Analyses of repeated cognitive testing examined whether infections linked to brain atrophy were associated with longitudinal performance across five cognitive domains. The interaction between infection history and infection-related brain atrophy on rates of cognitive decline was examined. **e** Analyses examined whether infections linked to brain atrophy were associated with elevated PET-defined cortical Aβ levels (Aβ +), and differences in baseline and longitudinal trajectories of ADRD plasma biomarkers. The interaction between infection history and infection-related brain atrophy on ADRD plasma biomarker was also examined. Key: Aβ_42/40_, amyloid-beta ratio; GFAP, glial fibrillary acidic protein; ICD, International Classifications of Diseases; MRI, magnetic resonance imaging; NfL, neurofilament light chain; pTau-181, phosphorylated tau-181

The present study’s primary analyses utilized data from the BLSA (Fig. 1), a continually enrolling study initiated in 1958 that is designed to assess physical and cognitive measures in a cohort of community-dwelling volunteers [12]. Participants received comprehensive health and functional screening evaluations at each study visit (including medical diagnostic code documentation, blood draws, cognitive and physical examinations etc.,), which were completed by licensed health-care professionals (e.g., nurse practitioner, medical doctor). Study visits occurred biennially until 2005, then every 1 to 4 years depending on age (age < 60 years, every 4 years; age 60–79 years, every 2 years; age ≥ 80 years, every year). For a subset of participants enrolled in the BLSA neuroimaging sub-study, visits occurred annually beginning in 1994. Due to BLSA’s continuous enrollment, participants entered the study at different times and varied with respect to follow-up times. Participants were selected for the present analyses if they had International Classification of Diseases, Ninth Revision (ICD9) medical codes, and MRI, cognitive performance, PET, or Simoa biomarker data. Participants were excluded based on missing covariate data, a history of neurologic conditions that could affect brain structure or function (e.g., strokes, seizures) and cognitive impairment at baseline. The current manuscript follows Strengthening the Reporting of Observational Studies in Epidemiology (STROBE) reporting guidelines.

### Infection diagnoses

The current study identified infections through the use of medical diagnostic codes, a strategy that has been applied previously [1, 5, 13]. Participants were categorized according to the presence (+) or absence (-) of an infection using infection-related medical codes derived from a recent study in the Danish National Patient Registry [13] along with ICD codes documented at enrollment and each BLSA study visit. Infections were classified a priori into fifteen categories including influenza, pneumonia, tuberculosis, candidiasis/fungal, miscellaneous bacterial infections (e.g., *Bordetella pertussis*, *Staphylococcus aureus, *etc*.,*), gastrointestinal infections, sexually transmitted infections, human herpes virus infections, viral hepatitis, miscellaneous viral infections, upper respiratory tract infections (URTIs), lower respiratory tract infections, skin and subcutaneous infections, urinary tract infections (UTIs), and ‘other’ infections; infection categories with limited numbers of participants (< 20 cases; e.g., HIV) were not considered (Supplementary Table 1). ICD9 codes in BLSA were derived from one or more potential sources of information collected at enrollment and each study visit, including laboratory test results, medical history records, and comprehensive health and functional screening evaluations at each study visit. Evidence of infections were adjudicated by a central panel of board-certified clinicians to ensure diagnostic reliability and accuracy. Such classification parallels previous methodology in that the diagnosed sample primarily represents infected participants with clinical symptoms severe enough to be detected during patient-health care consultations [4, 14]. We note that this infection classification likely lacks sensitivity to detect all infections, particularly asymptomatic cases. Given the link between bacterial/virial loads and symptom presentation, as well as the collateral consequences of the microbial and inflammatory exposure on host cells and tissues, we suspect that the infections identified here likely have a greater potential – compared to unascertained infections – to contribute to aberrant neurobiological processes. Participants with infection diagnoses documented at or before study enrollment or during follow-up visits (i.e., a participant’s infection occurred in-between study visits) were considered exposed. For positive and negative cases, the baseline visit for each participant was the earliest visit at which the presence or absence of an infection diagnosis was documented, respectively.

### 3 T brain MRI

T1-weighted magnetization-prepared rapid gradient echo (MPRAGE) scans were acquired on a 3 T Philips Achieva (repetition time [TR] = 6.8 ms, echo time [TE] = 3.2 ms, flip angle = 8°, image matrix = 256 × 256, 170 slices, pixel size = 1 × 1 mm, slice thickness = 1.2 mm, sagittal acquisition) beginning in 2008–2010. 3 T-MRI data were collected at baseline and follow up visits. A validated, Multi-atlas Region Segmentation Utilizing Ensembles (MUSE) anatomic labelling method specifically designed to achieve a consistent parcellation of brain anatomy in longitudinal MRI studies using T1-weighted sequences was applied [15]. Using volumes of MUSE-segmented brain regions as input features, we applied a semi-supervised deep representation learning approach (Surreal-GAN) to calculate R indices (R1, R2, R3, R4, R5). In brief, this approach uses neuroimaging data to learn functions for transforming a reference group to a target group through the generative adversarial network. The resulting latent variables encapsulate heterogeneous brain volume patterns mapped between younger (< 50 years old) and older (> 50 years old) adults, resulting in five continuous, low-dimensional scores that reflect the co-expression level of respective brain atrophy dimensions, and account for simultaneous spatial and temporal disease heterogeneity within the same individual. These scores have been pretrained and validated in a diverse multicohort dataset across 11 studies (> 49,000 participants), where they predict age-related clinical traits and disease diagnoses, and then applied to the current dataset [9].

### Cognitive impairment

Cognitive status was adjudicated as detailed previously [16]. In brief, participant serial clinical and neuropsychological data were reviewed at each consensus case conference if the participant had > 3 errors on the Blessed Information-Memory-Concentration test, or ≥ 0.5 total combined score on the Clinical Dementia Rating Scale. MCI was based on Petersen’s criteria, and dementia was based on the criteria outlined in the Diagnostic and Statistical Manual of Mental Disorders, third edition revised, and the National Institute of Neurological and Communicative Disorders and Stroke-Alzheimer's Disease and Related Disorders Association.

### Cognitive performance

Composite scores across five cognitive domains (visuospatial ability, verbal memory, verbal fluency, executive functioning, attention) were calculated from standardized (converted to a z-score using the baseline mean and SD) and averaged individual task components, as described previously [4]. Cognitive performance was measured at baseline and follow up visits. As certain cognitive tasks were initiated in the BLSA at different periods according to protocol changes, composite scores for participants at each visit were computed from those tasks available at the time of assessment. Visuospatial ability was assessed using a modified version of the Educational Testing Service Card Rotations Test and two Clock Drawing Tests (CDTs), where participants were asked to draw the hands and face of clocks indicating 3:25 and 11:10. Here, a composite score was calculated using the average of the standardized z-scores from the Card Rotations Test and the mean of the CDTs. Verbal memory was assessed using immediate (sum of 5 learning trials) and long-delay free recall from the California Verbal Learning Test. Verbal fluency was calculated using Verbal Fluency-Letters (F, A, S) and Verbal Fluency-Categories (fruits, animals, vegetables). Executive function was assessed using Trail Making Test Part B and the Digit Span Backward subtest of the Wechsler Adult Intelligence Scale-Revised. Attention was evaluated using Trail Making Test Part A and the Digit Span Forward subtest of the Wechsler Adult Intelligence Scale-Revised. Scores of Trail Making Test Part A and Part B were first natural log transformed, z scored, and then signs inverted, so that higher scores reflect higher performance (i.e., consistent with the direction of performance across other cognitive tasks).

### Alzheimer’s disease and neurodegeneration biomarkers

Aβ_40_, Aβ_42_, GFAP, NfL, and pTau-181 concentrations were measured using the Single Molecule Array (Simoa) Neurology 4-Plex E (N4PE) and pTau-181 (V2) assays on the Simoa HD-X instrument (Quanterix). Plasma used for analyses was collected at baseline and follow up visits. Unlike NfL and GFAP, Aβ_42/40_ and pTau-181 are more specific to AD and the amyloid cascade. Assays were run in duplicate, and values averaged. CVs were 1.5, 1.0, 4.9, 4.8, and 4.4% for Aβ_40_, Aβ_42_, GFAP, NfL, and pTau-181, respectively. Values for GFAP, NfL, and pTau-181 were log_2_ transformed. Aβ_42/40_ ratio and standardized values were used in analyses.

### PET

^11^C-Pittsburgh compound-B (PiB) distribution volume ratios (DVR) were measured using Aβ PET [17]. PET scans (70 min.) were collected at baseline on a GE Advance or Siemens High Resolution Research Tomograph scanner immediately following an i.v. bolus injection of approximately 555 MBq of the radiotracer. DVRs were computed with a spatially constrained simplified reference tissue model using cerebellar gray matter as a reference. Mean cortical Aβ reflected the average DVR values across the cingulate, frontal, parietal (including precuneus), lateral temporal, and lateral occipital regions, excluding the pre- and post-central gyri. Mean cortical DVR values were harmonized between the two scanners by leveraging longitudinal data available on both scanners for 79 participants. Aβ PET status (±) was defined based on a Gaussian mixture model threshold of 1.064 mean cortical DVR [17].

### Covariates

Baseline age (years), sex (male/female), race (white/non-white), and education level (years) were defined based on participant self-reports. *APOE*ε4 carrier status (0 ε4 alleles/≥ 1 ε4 alleles/missing) was defined via PCR with restriction isotyping using the Type IIP enzyme Hhai or the Taqman method. Estimated glomerular filtration rate (eGFR)-creatinine was defined at the time of blood sample collection using the CKD-EPI criteria. Comorbid diseases which represent potential confounders were defined using a comorbidity index calculated as the sum (score range: 0–8; converted to a percentage to account for missing data) of eight conditions: obesity, hypertension, diabetes, cancer, ischemic heart disease, chronic heart failure, chronic kidney disease and chronic obstructive pulmonary disease [4].

### UK Biobank (UKB)

Exposure to infections was ascertained from primary and secondary diagnoses in linked hospital discharge records from HES APC (Hospital Episode Statistics–Admitted Patient Care), SMR01 (Scottish Morbidity Records–General/Acute Inpatient and Day Case Admissions), and PEDW (Patient Episode Database for Wales) with ICD9 and ICD10 medical codes corresponding to the infection-related ICD9 codes used in BLSA. The exclusive reliance on discharge records may account for the limited number some infections cases (e.g., influenza) ascertained in the current UKB longitudinal neuroimaging sample. T1-weighted MPRAGE scans were acquired on a 3 T Siemens Skyra, as described previously [18]. Machine learning-derived neuroimaging measures of age-related brain atrophy were generated using the same methodology as described in the BLSA. Covariate data were collected at study enrollment except for age, which was derived from the time of first brain scan. Covariates included age, sex, socioeconomic status (low, intermediate, high), body mass index (< 18.5, 18.5–24.9, 25.0–29.9, ≥ 30 kg/m^2^), hypertension, alcohol consumption (never, former, 3 classes of moderate, intermediate, heavy), smoking (never, former, current) and diabetes. Socioeconomic status was based on self-reported educational attainment: low (no qualification), high (college or university degree), or intermediate (all others). *APOE*ε4 carrier status was based on two single-nucleotide polymorphisms, rs429358 and rs7412, that were directly genotyped using UK Biobank Axiom array.

### Statistical analyses

To align with our primary research objective (i.e., how infections might impact longitudinal changes in brain structure), and secondary research objectives (i.e., whether such infection-related brain atrophy can influence cognitive decline and molecular changes underlying dementia), a hierarchical gating approach was employed, whereby infections of interest for downstream analyses were first identified with discovery analyses of infection-related brain volume loss in the BLSA using a nominally significant p-value of 0.05 (although FDR-corrected results are also presented) [19]. To reduce the possibility of Type I error, we employed an external cohort (i.e., the UKB) to validate infection-related brain atrophy associations, as well as multiple orthogonal approaches to support associations between infection history and the emergence of ADRD endophenotypes. Linear mixed effects models were used to examine associations of infections with longitudinal rates of change in neuroimaging signatures, cognitive performance, and ADRD biomarkers. Models included the following covariates: baseline age, sex, race, education, *APOE*ε4, comorbidity index, and the interactions of age, sex, race, education, *APOE*ε4, and comorbidity index with time. ADRD biomarker analyses also adjusted for eGFR-creatinine. Intracranial volume (icv) was not adjusted for in neuroimaging analyses because icv-residualized values were used in initial calculations of the atrophy measures. Random effects of intercept and time with unstructured covariance were included to account for the within-subject correlation. The main fixed effect of interest was infection*time, which estimated the effect of infection on rates of change. Thus, infection was treated as a fixed predictor variable, whereas neuroimaging signatures, cognitive performance, and ADRD biomarkers were treated as time-varying outcome variables. Specifically, the fixed infection term reflected a participant’s infection status at the time of baseline (i.e., time zero) MRI scan, cognitive assessment, and/or blood draw for longitudinal analyses in which brain atrophy, cognitive performance, or ADRD biomarkers, respectively, were time-varying outcome variables. Separate linear mixed effects models incorporated two-way and three-way interaction terms (infection*brain atrophy, infection*brain atrophy *time) to examine whether rates of brain atrophy modified the associations of infections with longitudinal cognitive performance and ADRD biomarker changes. Logistic regression models adjusted for the aforementioned covariates were used to examine associations of infections with cognitive status. Insufficient numbers of incident cognitive impairment cases in the current dataset prevented the application of other statistical approaches, such as time-to-event analyses. Logistic regression models adjusted for age, sex, *APOE*ε4, and comorbidity index were used to examine associations of infections with Aβ PET status (±). In the UKB, linear mixed effects models adjusted for baseline age, age squared, sex, socioeconomic status, *APOE*ε4, BMI, hypertension, diabetes, alcohol consumption, smoking, and two-way interactions of covariates with time were used to examine associations of infections with longitudinal rates of change in neuroimaging signatures. Statistical significance was defined at two-sided p < 0.05. FDR correction was applied for each infection. Model quality and goodness of fit (e.g., overdispersion, singularity, heteroskedasticity etc.,) were assessed using the performance R package (0.11.0.8). Model assumptions were satisfied and normal distributions of outcomes were verified with Kolmogorov–Smirnov tests (i.e., all p-values > 0.05) and visual inspection via histograms. Analyses were performed using R (4.2.2) and Stata/MP [18]. Graphs were generated in R and the Biorender platform (https://www.biorender.com).

## Results

### Infections are related to accelerated brain atrophy

Infections were classified a priori into fifteen categories using ICD medical diagnostic codes, and included influenza, pneumonia, tuberculosis, candidiasis/fungal, miscellaneous bacterial infections (e.g., *Bordetella pertussis*, *Staphylococcus aureus, *etc*.,*), gastrointestinal infections, sexually transmitted infections, human herpes virus infections, viral hepatitis, miscellaneous viral infections, upper respiratory tract infections (URTIs), lower respiratory tract infections, skin and subcutaneous infections, urinary tract infections (UTIs), and ‘other’ infections (Fig. 1; Supplementary Table 1). We asked if participants with a history of specific infections showed longitudinal changes in five (R1-R5) machine learning measures of age-related atrophy, which primarily reflect atrophy in subcortical (R1), medial-temporal lobe (R2), parieto-temporal lobe (R3), diffuse cortical (R4), and perisylvian (R5) regions. These measures have been developed and validated in large external cohorts, where they predict age-related clinical traits, cognitive decline, and neurodegenerative disease risk [20]. A total of 793 cognitively normal BLSA participants with at least one 3 T-MRI brain scan were eligible for analyses (age = 70.1 yrs. [SD = 9.8]; 55.6% female; 66.5% white; Supplementary Fig. 1; Supplementary Table 2). The average time between the date of any infection diagnosis and baseline scan was 16.5 years (median: 14.2, IQR: 8.8, 25.0) (Supplementary Table 3). For the 548 participants with multiple scans, the average follow-up time for was 5.4 years (median: 5.2, IQR: 3.9, 7.1) with an average of 3.7 (SD = 1.5) scans per participant (range: 2 to 11). Among participants with and without infections, mean follow-up times were similar. 63.1% of participants exhibited a history of any infection, and 12.1% exhibited a history of two or more infections.

**Fig. 2:**
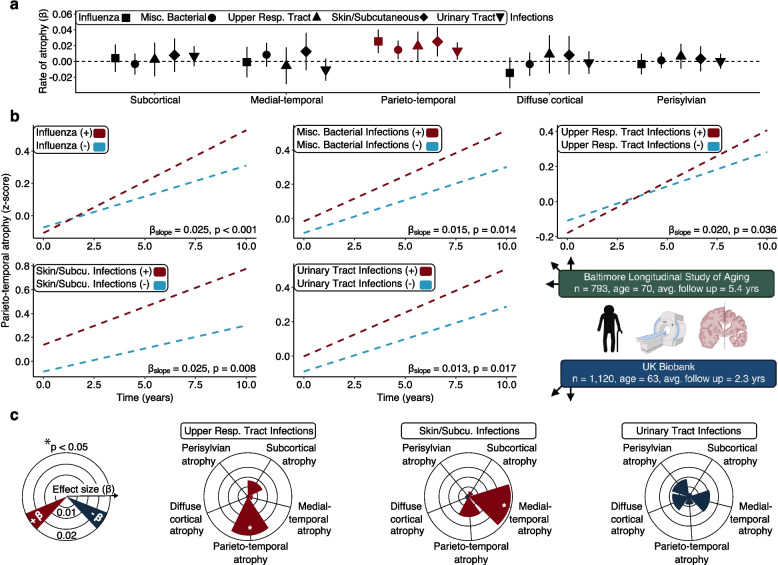
Infections are associated with annual changes in machine learning-derived neuroimaging signatures. **a** Forest plot shows the associations of influenza, miscellaneous bacterial, upper respiratory tract, skin/subcutaneous, and urinary tract infections with rates of change in neuroimaging signatures that capture distinct patterns of age-related brain volume loss (i.e., subcortical, medial-temporal lobe, parieto-temporal lobe, diffuse cortical, and perisylvian). Higher values reflect greater atrophy. Annual changes of standardized brain atrophy signatures associated with history of a given infection (β) were derived from linear mixed effects models adjusted for demographic, physiological, and comorbid variables, and two-way interactions of covariates with time. Red shapes indicate statistical significance (*p* < 0.05). **b** Scatterplots show parieto-temporal volume loss associated with influenza, miscellaneous bacterial, upper respiratory tract, skin/subcutaneous, and urinary tract infections. Annual changes of standardized brain atrophy signatures associated with history of a given infection (β) were derived from linear mixed effects models adjusted for the aforementioned covariates. **c** Rose plots show the rates of change in machine learning-derived neuroimaging signatures associated with upper respiratory tract, skin/subcutaneous, and urinary tract infections in an independent replication cohort (the UK Biobank; UKB). Insufficient cases prevented examination of influenza and miscellaneous bacterial infections. Annual changes of standardized brain atrophy signatures associated with history of a given infection (β) were derived from linear mixed effects models adjusted for demographic, physiological, and comorbid variables, and two-way interactions of covariates with time

Of the fifteen infections examined, five (influenza [β = 0.03, *p* < 0.001, FDR *p* = 0.004], URTIs [β = 0.02, *p* = 0.036, FDR  = 0.180], miscellaneous bacterial [β = 0.01, *p* = 0.014, FDR *p* = 0.070], skin/subcutaneous [β = 0.03, *p *= 0.008, FDR *p* = 0.040], and UTIs [β = 0.01, *p* = 0.017, FDR *p* = 0.087]) were associated with accelerated parieto-temporal atrophy at an uncorrected p < 0.05 in linear mixed effects models adjusted for demographic, physiological, and comorbid variables (Fig. 2A, B; Supplementary Table 4). The average annual effects of these five infections on parieto-temporal atrophy (infection*time average β = 0.02; minimum β = 0.01 [UTIs]; maximum β = 0.03 [influenza]) were equivalent to 20 years of additional age (separate model; age*time β = 0.001, p < 0.001). Other than detecting greater diffuse cortical atrophy associated with UTIs, infections were unrelated to baseline differences in brain structure (Supplementary Table 4). The associations of influenza and skin/subcutaneous infections with accelerated parieto-temporal atrophy survived FDR correction, and results were similar in sensitivity analyses examining effect modification by self-reported sex. These five infections were not related to changes in other neuroimaging signatures, and no other infections were related to changes in brain structure (including a history of any infection and total frequency of infections).

To validate the associations we identified in the BLSA, we examined if influenza, URTIs, miscellaneous bacterial, skin/subcutaneous, and UTIs were related to changes in the same machine learning-derived neuroimaging signatures measured in an external cohort, the UKB, using similar co-variate adjusted linear mixed effects models. A total of 1,120 participants were included in these analyses (age = 62.9 yrs. [SD = 7.2]; 51.5% female; 97.8% white; Supplementary Table 5), where participants had two MRI scans over an average 2.3 (SD = 0.1) years. Although insufficient cases (n < 5) prevented examination of influenza and miscellaneous bacterial infections in this cohort, we found that a history of URTIs predicted greater parieto-temporal atrophy (β = 0.02, *p* = 0.033), whereas a history of skin/subcutaneous infections was associated with accelerated medial-temporal lobe atrophy (β = 0.02, *p* = 0.007; Fig. 2C; Supplementary Table 6). Notably, the limited number of cases in the UKB reduced our power to detect statistically significant effects. These findings suggest that participants with a history of certain infections are susceptible to accelerated brain volume loss over time, particularly in parieto-temporal regions.

### Infection history is associated with higher odds of dementia

**Fig. 3:**
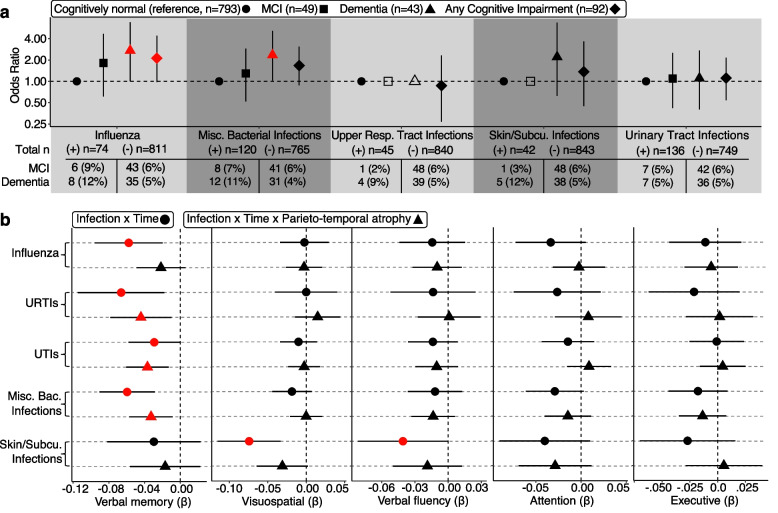
Infections are associated with cognitive impairment and longitudinal cognitive performance. **a** The odds of prevalent cognitive impairment associated with a history influenza, miscellaneous bacterial, upper respiratory tract, skin/subcutaneous, and urinary tract infections. Odds of mild cognitive impairment, dementia, and any form of cognitive impairment compared to cognitively normal status (Odds Ratio) were derived from logistic regression models adjusted for demographic, physiological, and comorbid variables. Orange shapes indicate statistical significance (*p* < 0.05). Hollow shapes indicate insufficient cases were available for analyses. The percentages indicated in the accompanying table reflect frequencies used in analyses (e.g., mild cognitive impairment vs cognitively normal). **b** Forest plots show the rates of domain-specific cognitive decline associated with influenza, miscellaneous bacterial, upper respiratory tract, skin/subcutaneous, and urinary tract infections, and how infection-related atrophy (infection*parieto-temporal atrophy*time) moderates this association. Annual changes in cognition associated with history of a given infection (β) were derived from linear mixed effects models adjusted for the aforementioned covariates. Orange shapes indicate statistical significance (*p* < 0.05). Key: MCI, mild cognitive impairment; URTIs, upper respiratory tract infections; UTIs, urinary tract infections

Leveraging data from cognitively unimpaired (*n* = 793) BLSA participants who were included the preceding analyses, along with data from BLSA participants who were otherwise excluded from analyses due to MCI (*n* = 49) and dementia (*n* = 43), we determined whether the same infections linked to brain atrophy were also associated with cognitive impairment using logistic regression models adjusted for demographic, physiological and comorbid variables. On average, 14.3% of participants with a history of influenza, URTIs, miscellaneous bacterial, skin/subcutaneous, and UTIs displayed some form cognitive impairment, whereas 10.0% of uninfected participants were cognitively impaired. Although insufficient cases (< 5) prevented examination of several associations (e.g., MCI risk with URTIs and skin/subcutaneous infections), we detected increased odds of dementia and any cognitive impairment among participants with a history of influenza infections (dementia odds ratio [OR] = 2.72, *p* = 0.038; any cognitive impairment OR = 2.12,* p* = 0.049), as well as increased odds of dementia among participants with a history of miscellaneous bacterial infections (OR = 2.35, *p* = 0.036; Fig. 3A; Supplementary Table 7).

### Infections and infection-related brain atrophy are associated with accelerated cognitive decline

Next, we examined the associations of influenza, URTIs, miscellaneous bacterial, skin/subcutaneous, and UTIs with annual rates of change across five cognitive domains (visuospatial abilities, verbal fluency, verbal memory, executive function, and attention) using linear mixed effects models adjusted for demographic, physiological and comorbid variables. Additionally, we determined whether patterns of brain atrophy most strongly associated with infection history (i.e., parieto-temporal atrophy) influenced the extent of infection-related cognitive declines (i.e., infection*brain atrophy*time) using linear mixed effects models adjusted for the aforementioned covariates. Analyses included 548 cognitively normal BLSA participants who had longitudinal neuroimaging and cognitive data (age = 72.4 yrs. [SD = 9.2]; 56.8% female; 66.8% white; Supplementary Table 8).

A history of miscellaneous bacterial (β = −0.06, *p* < 0.001), URTIs (β = −0.07, *p* = 0.007), and UTIs (β = −0.03, *p* = 0.049) were associated with greater reductions in verbal memory scores, and parieto-temporal atrophy was associated with an acceleration in these declines (all interactions* p* < 0.05; Fig. 3B; Supplementary Table 9). Influenza was also associated with greater reductions in verbal memory (β = −0.06, *p* = 0.003), and skin/subcutaneous infections were related to accelerated declines in both visuospatial abilities (β = −0.07, *p* < 0.001) and verbal fluency (β = −0.04, *p* = 0.049), but parieto-temporal atrophy did not modify these effects. No infections were related to trajectories of executive function or attention domains. These results indicate that i) participants with a history of certain infections display accelerated rates of cognitive decline in specific domains, and ii) infection-related verbal memory declines are more prominent among individuals who experience greater post-infection parieto-temporal brain atrophy.

### Infections and infection-related brain atrophy are associated with higher ADRD biomarkers

We also investigated the effects of influenza, URTIs, miscellaneous bacterial, skin/subcutaneous, and UTIs on PET-defined amyloid-positive status (Aβ +), baseline differences in ADRD plasma biomarkers, and changes in plasma biomarkers over time using logistic and linear mixed effects models adjusted for demographic, physiological and comorbid variables. Additionally, we examined whether atrophy in brain regions vulnerable to infection (i.e., parieto-temporal atrophy) altered the relationships of infection history with ADRD plasma biomarker trajectories. Plasma biomarker analyses included longitudinal neuroimaging and biomarker data from cognitively normal BLSA participants (amyloid-beta ratio, Aβ_42/40_; neurofilament light chain, NfL; glial fibrillary acidic protein, GFAP; *n *= 548; phosphorylated tau-181, pTau-181; *n* = 477; Supplementary Table 10). A subset of BLSA participants (*n* = 157) was included in Aβ PET analyses (Aβ + *n* = 45, Aβ- *n* = 112).

**Fig. 4:**
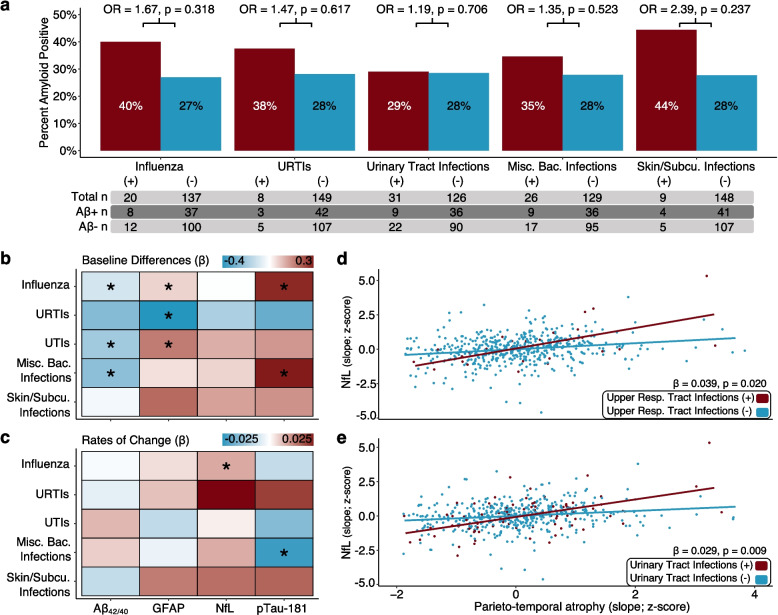
Infections are associated with biomarkers of Alzheimer’s disease and related dementias (ADRD). **a** The percentages (and corresponding frequencies) of amyloid-β (Aβ) positive (+) and negative (-) participants who maintain a history of influenza, miscellaneous bacterial, upper respiratory tract, skin/subcutaneous, and urinary tract infections. Odds of Aβ + (Odds Ratio) were derived from logistic regression models adjusted for demographic, physiological, and comorbid variables. Heatmaps show the **b** baseline and **c** longitudinal associations of infections with plasma biomarker levels. Differences and annual changes in plasma biomarker levels associated with history of a given infection (β) were derived from linear mixed effects models adjusted for demographic, physiological, and comorbid variables, and two-way interactions of covariates with time. Scatterplots show how a history of **d** upper respiratory tract **e** and urinary tract infections modifies the longitudinal associations of infection-related atrophy (i.e., parieto-temporal atrophy) with plasma NfL levels (infection*parieto-temporal atrophy*time). Annual changes in NfL levels associated with history of a given infection (β) were derived from linear mixed effects models adjusted for the aforementioned covariates. Individual-specific standardized slopes of NfL and parieto-temporal atrophy are displayed. Key: Aβ_42/40_, amyloid-beta ratio; ADRD, Alzheimer’s disease and related dementias; GFAP, glial fibrillary acidic protein; NfL, neurofilament light chain; OR, odds ratio; pTau-181, phosphorylated tau-181; UKB, UK Biobank; URTIs, upper respiratory tract infections; UTIs, urinary tract infections

Although nonsignificant (potentially due to limited statistical power), each infection was related to a higher likelihood of Aβ +, ranging from 19% greater odds of Aβ + for UTIs to 139% greater odds of Aβ + for skin/subcutaneous infections (Fig. 4A; Supplementary Table 11). Consistent with these suggestive PET findings, three of the five infections of interest were associated with significantly lower plasma levels of Aβ_42/40_ (indicative of higher brain Aβ), including influenza (β = −0.01, *p* = 0.011; also related to higher pTau-181 [β = 0.36, *p* = 0.018] and GFAP [β = 0.07, *p* = 0.035]), miscellaneous bacterial infections (β = −0.24, *p* = 0.029; also related to higher pTau-181 [β = 0.38, *p* = 0.003]) and UTIs (β = −0.20, *p* = 0.043; also related to higher GFAP [β = 0.22, *p* = 0.015]; Fig. 4B; Supplementary Table 11). We also detected an association between URTIs and lower GFAP (β = −0.40, *p* = 0.009). In longitudinal analyses, influenza was associated with accelerated increases in NfL (β = 0.02, *p* < 0.001), and miscellaneous bacterial infections were related to attenuated increases in pTau-181 (β = −0.05, *p* = 0.010; Fig. 4C; Supplementary Table 11). Parieto-temporal atrophy was associated with accelerated increases in NfL (β = 0.02, *p* < 0.001), supporting this biomarker’s role as an indicator of neurodegeneration (Supplementary Table 11). Although URTIs and UTIs alone were not related to changes in plasma biomarkers, the increases in NfL associated with parieto-temporal atrophy were accelerated among participants with a history of URTIs (β = 0.04, *p* = 0.020) and UTIs (β = 0.03, *p* = 0.009; Fig. 4D, E; Supplementary Table 11). These findings suggest that participants with a history of certain infections show higher levels of brain Aβ (i.e., lower plasma levels of Aβ_42/40_) and other plasma ADRD biomarkers, while infections and post-infection brain atrophy are related to increases in a non-specific marker of neurodegeneration.

## Discussion

**Fig. 5:**
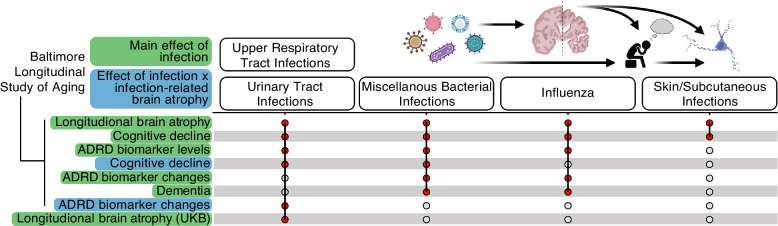
A modified upset plot summarizes results from the current study. The x-axis represents infection types and the y-axis represents outcomes examined in the Baltimore Longitudinal Study of Aging (BLSA) and UK Biobank (UKB). Horizonal white and gray bars improve interpretability of the y-axis outcomes across x-axis infection types. Orange circles reflect significant associations detected in statistical analyses, with the intersecting black lines highlighting such associations according to each infection type. The main effect of infection (highlighted in green) represents observed associations when a given infection is used as the predictor variable (e.g., influenza). The effect of infection x infection related-brain atrophy (highlighted in blue) represents observed associations when the interaction of a given infection and infection-related brain atrophy (i.e., parieto-temporal atrophy) is used as the predictor variable (e.g., influenza*parieto-temporal atrophy)

Using a community-based cohort of older adults with machine learning-derived neuroimaging signatures obtained from longitudinal 3 T-MRI, the current study demonstrated that a history of influenza, upper respiratory tract (URTIs), miscellaneous bacterial, skin/subcutaneous, and urinary tract infections (UTIs) were associated with accelerated parieto-temporal atrophy. After illustrating their relationships with prevalent cognitive impairment and longitudinal neuroimaging signatures in an independent cohort, we showed these infections – especially in the context of parieto-temporal atrophy – can accelerate cognitive decline (particularly verbal memory) and ADRD plasma biomarker trajectories (particularly NfL). The present findings support the role of infections in mediating clinically relevant brain atrophy patterns, highlight the potential consequences of post-infection brain volume loss on longitudinal neurocognitive outcomes, and extend our understanding of the biological basis by which infectious pathogens may contribute to neurodegeneration and ultimately increase risk for dementia (Fig. 5).

The accelerated rates of brain atrophy associated with infections in our study are consistent with reports of brain volume loss related to SARS-CoV-2 and HIV infections in other cohorts [3, 7]. With an average of over 16 years between a first report of infection and baseline MRI scan, we suspect the observed differences in brain structure may differ greatly from those seen in the acute or early post-acute phase of an infection. Although baseline differences were not detected in our analyses, we hypothesize that lower brain volumes and greater rates of atrophy could be evident if infections occurred (on average) closer to MRI acquisition, especially if such exposures resulted in hospitalization. The infection-related atrophy in our analyses may be attributed to long-term immune activation and systemic inflammation associated with prior infections or host immune traits of infection susceptibility and maladaptive inflammatory responses that consequently facilitate neurodegeneration [21]. Thus, infection history (or vulnerability to infections) may be functioning as a proxy for other long-term processes (i.e., systemic inflammation, immune dysfunction etc.,) that contribute to, or accompany, neurodegenerative pathophysiology. This postulation is supported by a recent report of elevated dementia risk among individuals who did not receive herpes zoster vaccination [22]. Additional investigations in other community-based cohorts will help determine whether the atrophy patterns observed in our study represent long-term consequences of infections, or reflect differences in underlying health, immune vulnerability, or survivorship among participants with documented infections.

We were unable to replicate all infection-brain atrophy associations observed in the BLSA within the UKB, and in some cases the implicated patterns of atrophy differed (i.e., skin/subcutaneous infections were associated with accelerated parieto-temporal atrophy in BLSA but accelerated medial-temporal lobe atrophy in UKB). This may be attributed to several factors, including differences in study design (average follow up of 5.4 years, 3.7 scans in BLSA versus average follow up of 2.3 years, 2.0 scans in UKB), key demographic characteristics of the participants (mean age 70 years old, 67% white in BLSA versus mean age 63 years old, 98% white in UKB), scanner/methodological differences (i.e., Philips Achieva in BLSA and Siemens Skyra in UKB), or other idiosyncrasies (e.g., ICD sources for infection ascertainment). Although the vulnerability of parieto-temporal structures aligns with our prior data showing infection-related volume loss in the temporal lobe [5], the absence of infection-related changes in other neuroimaging signatures may be due to regional differences in brain-resident or infiltrating immune cells, varying tropism of different pathogens across brain regions and cell types, and differing systemic exposures via alternative neurovasculature [23–27]. The unexpected associations we observed in some of our exploratory biomarker analyses (i.e., lower GFAP with URTIs, attenuated pTau-181 increases with miscellaneous bacterial infections) should be interpreted with caution, but could reflect the temporally dynamic and pleiotropic relationships between inflammation and neurodegeneration that can vary dependent on disease stage [28, 29].

Given that i) parieto-temporal atrophy is itself characteristic of AD [30], ii) the parieto-temporal neuroimaging signature used in our study predicts cognitive status as well as MCI to dementia progression in external cohorts [20], iii) each of the examined infections was related to accelerated cognitive decline (especially verbal memory), iv) a majority of infections were associated with higher plasma biomarker levels at baseline, and v) a subset of infections were related to higher odds of dementia and accelerated increases in a non-specific marker of neurodegeneration (NfL), our study presents compelling evidence that even distal infectious exposures may accelerate the emergence of ADRD endophenotypes in older adults. As noted above, the two unexpected associations with plasma biomarkers (i.e., lower GFAP with URTIs and slower pTau-181 increases with miscellaneous bacterial infections) may be attributed to a variety of factors, including the antimicrobial properties of pathological proteins underlying ADRD [31, 32], as well as the pleiotropic and disease stage-dependent relationships between immune functioning and AD neuropathology [33–35].

The current study integrates complementary neurocognitive outcomes within a community-based sample of deeply-phenotyped older adults, employs neuroimaging data from an independent cohort, and applies advanced machine-learning techniques to deconvolute the longitudinal consequences of infections on neurodegeneration. However, these results should be interpreted within the context of the study’s limitations. The reliance on medical diagnostic codes to identify infections limits the generalizability of our findings, given an inability to identify subclinical or asymptomatic individuals whose infection status is not detected during patient-health care consultations and who might otherwise be identified with serology, polymerase chain reaction, and/or antigen assays. Despite the inherently exploratory nature of the current study, we acknowledge that the number of comparisons in our primary analyses of infection-related brain volume loss had the potential to inflate Type I error, and the limited number of cases for certain infections and outcomes (e.g., Aβ PET in BLSA, MRI replication in UKB, etc.,) reduced power to detect statistically significant effects. Therefore, while we did not use multiple comparison-adjusted p values to determine statistical significance in our primary analyses, we employed multiple methods and data sources to study our research questions (i.e., triangulation). Because of the retrospective nature of our study, we were able to precisely identify when an infection was documented at BLSA study visits, but not the exact date when the infection occurred (i.e., an infection could have occurred at any time preceding enrollment or in between study visits); as this prevented the reliable calculation of a time-since-infection variable that would be valid for statistical analyses, we were unable to control for the time between a participant’s infection and baseline MRI scan or determine whether age at infection or time since infection modified the relationship between infection and neurocognitive outcomes. Despite these limitations, our findings suggest an association between infections and accelerated rates of regionally specific brain atrophy, while highlighting the consequences of such infection-related atrophy on cognitive decline and molecular signatures of ADRD.

## Supplementary Information

Supplementary Material 1

Supplementary Material 2

## Data Availability

All data generated in the current study are included in this article and its supplementary materials, available upon reasonable request from an independent steering committee, or available in an online public repository. Researchers who wish to use BLSA data are encouraged to develop a pre-analysis plan that can be submitted for approval (https://blsa.nia.nih.gov/how-apply). Data, protocols, and other metadata of the UK Biobank are available to the scientific community upon request in accordance with the UK Biobank data sharing policy (https://www.ukbiobank.ac.uk/enable-your-research/apply-for-access).
