# Supplementary figures and images for: Post-infection brain atrophy accelerates cognitive and molecular changes underlying dementia
Source: Alzheimers Res Ther. 2025 Dec 5;18:8. doi: 10.1186/s13195-025-01924-2 (PMC12797566; doi:10.1186/s13195-025-01924-2)

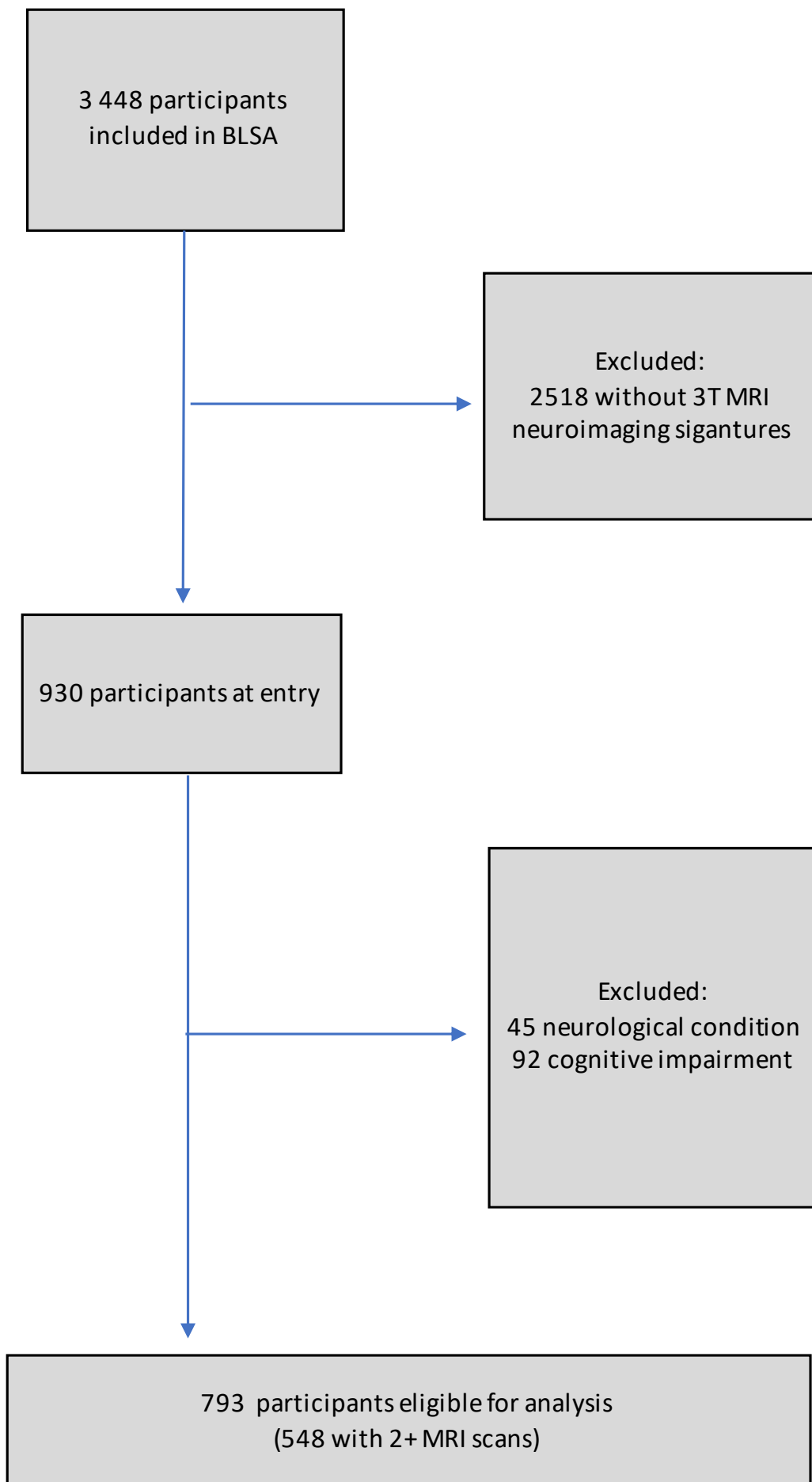

**Supplementary Figure 1.** Flow chart of participant selection for analyses in the BLSA.

Supplement: Supplementary file 1 — Supplementary Material 1 [file 13195_2025_1924_MOESM1_ESM.pdf]
